# Self-perceived oral symptoms and periodontal inflammatory conditions in habitual *naswar* dippers

**DOI:** 10.12669/pjms.345.15418

**Published:** 2018

**Authors:** Nawwaf Al-Hamoudi, Sameer Mokeem, Tariq Abdul Jabbar, FahimVohra, Zohaib Akram

**Affiliations:** 1 Nawwaf Al-Hamoudi, Department of Periodontics and Community Dentistry, King Saud University, Riyadh, Saudi Arabia; 2 Sameer Mokeem, Department of Periodontics and Community Dentistry, King Saud University, Riyadh, Saudi Arabia; 3 Tariq Abdul Jabbar, Professor, Department of Prosthetic Dental Science, College of Dentistry, King Saud University; Engineer Abdullah Bugshan Research Chair for Growth Factors and Bone Regeneration, College of Dentistry, Riyadh 11545, Saudi Arabia; 4 Fahim Vohra, Professor, Department of Prosthetic Dental Science, College of Dentistry, King Saud University; Engineer Abdullah Bugshan Research Chair for Growth Factors and Bone Regeneration, College of Dentistry, Riyadh 11545, Saudi Arabia; 5 Zohaib Akram Department of Periodontology, Faculty of Dentistry, Ziauddin University, Karachi, Pakistan

**Keywords:** Alveolar bone loss, Inflammation, oral symptoms New tobacco products, Periodontitis

## Abstract

**Objectives:**

To compare self-perceived oral symptoms and clinical (plaque index [PI], bleeding on probing [BOP], clinical attachment loss [CAL]) and radiographic (marginal bone loss [MBL]) periodontal parameters among *naswar* (NW) and non-*naswar* dippers (NNW).

**Methods:**

One hundred and forty-two individuals (72 patients consuming *naswar* and 70 controls) were included. All participants completed a baseline questionnaire that included information regarding demographic characteristics and self-perceived oral symptoms. Clinical periodontal parameters (PI, BOP, PD and CAL) were recorded. MBL was measured on digital panoramic radiographs.

**Results:**

Pain in teeth, pain on chewing, bleeding gums and burning sensation in the mouth was significantly worse among NW than NNW (*p*<0.01). Clinical periodontal parameters and MBL were significantly high in NW than NNW (*p*<0.001). There was statistically significant influence of daily use and mean duration of *naswar* consumption on the severity of PI, BOP, PD (4 to 6 and >6 mm) and MBL among NW group.

**Conclusions:**

Self-perceived oral symptoms and periodontal parameters were worse among *naswar* dippers. It is highly recommended that *naswar* dipping should be considered a potential threat that could have major effects on periodontal tissues.

## INTRODUCTION

*Naswar* (also known as Nass or Niswar) is a type of dipping smokeless tobacco product (STP), made from fresh tobacco leaves, calcium oxide (slaked lime), wood ash and indigo widely consumed in Afghanistan, Iran, Pakistan, India and South Africa.^1^-^3^ *Naswar* is commercially available in transparent non-labeled pouches which are dispensed without a health threatening sign regarding its risk on oral and general health. *Naswar* is either used by ‘sniffing’ (nasally) or ‘dipping’ by placing in the buccal vestibule either in the upper or lower anterior or posterior area for 10–15 minutes.^1^,^2^ The contents are sucked intermittently and not chewed because of the bad taste. The contents are then spat out subsequently and never swallowed.

Previous studies have showed worse periodontal conditions such as plaque index (PI), bleeding on probing (BOP), probing depth (PD), clinical attachment loss (CAL) and marginal bone loss (MBL) in patients consuming *gutka* and betel quid.^4^-^6^ This could possibly be justified by the fact that the harmful constituents of *naswar* such as slaked lime, tobacco and areca nut prove to be detrimental and may be classified as independent risk factors for causing oral tissue inflammation. Slaked lime can enhance alkalinity in the oral environment that leads to the generation of reactive oxygen species (ROS) which favors oral mucosal inflammation and carcinogenesis whereas smokeless tobacco has been reported to cause hyperemia in gingival circulation.^7^,^8^

It is pertinent to mention that the contents have a high pH containing unionized nicotine and carcinogenic tobacco-specific N-*nitrosamines* (TSNAs), which have jeopardizing effects on oral and general health.^3^,^9^ We therefore hypothesized that self-perceived oral symptoms and clinical periodontal inflammatory parameters are worse in habitual *naswar* dippers (NW) as compared to patients not consuming *naswar* (NNW) as controls. Thus, the aim of the present study was to compare the self-perceived oral symptoms, clinical and radiographic periodontal parameters among NW and NNW controls.

## METHODS

This study was carried out in agreement with the Declaration of Helsinki. The study was reviewed and approved by the Research Ethics Review Committee of the College of Dentistry, Ziauddin University, Karachi, Pakistan. An informed consent was provided to all participants that explained the objectives and nature of the study protocol.

The study was performed from December 2015 to April 2016 at the Out-Patient Department, Ziauddin University, Karachi, Pakistan. In the present study, 142 individuals (72 patients consuming *naswar* (NW) and 70 controls (NNW) were included. The study participants were recruited from a local residential area in Karachi, Pakistan.

Only male individuals with ≥25 years of age who reported to consume ≥1packet of *naswar* daily since ≥ 1 year and individuals who reported to have never consumed *naswar* or any other tobacco product were included in the study. Self-reported systemic conditions that could affect periodontal structure, recent periodontal therapy, long-term administration of anti-inflammatory medication, habitual tobacco smoking or chewing and alcohol consumers were excluded.

All participants completed a structured baseline questionnaire that included the data regarding:

1. Age
2. Gender
3. Education status
4. Duration of *naswar* consumption
5. Daily frequency of *naswar* placement
6. Self-performed oral care including frequency of tooth brushing (once or twice).

All participants were also investigated about:

1. Bleeding gums
2. Pain on chewing
3. Dryness in mouth
4. Burning sensation in mouth based on dichotomous scoring system ‘yes’ or ‘no’.

Full-mouth periodontal assessment was carried out at six sites per tooth using North-Carolina-15 periodontal probe (Hu-Friedy, Chicago, IL, USA.) by a single calibrated examiner. (*Kappa*= 0.91). The following clinical parameters were assessed: full-mouth PI, BOP, PD (4 to 6 and >6 in mm) and CAL in mm on all teeth except third molars.^10^ The total number of missing teeth in the mouth was recorded for all the study participants.

Digital panoramic radiographs were taken using a panoramic tomography machine (KODAK 8000C System, Carestream Dental LLC, Atlanta, GA) and extrapolated on a calibrated computer screen (Samsung SyncMaster digital TV monitor, Korea).^11^,^12^ The data were analysed using a software program (Image Tool 3.0 Program, Department of Dental Diagnostic Science, University of Texas Health Science Center, San Antonio, TX).

Statistical analyses were carried out using statistical software (SPSS, v.20.0 for Windows, IBM, Chicago, IL). For all tests, *p*-value was set at 0.05. Normality of distribution of the variables was tested with Shapiro-Wilk tests. The significance of differences in periodontal parameters between the groups was determined using the Kruskal Wallis and Mann-Whitney U-tests. Odds ratios for self-perceived oral symptoms among NW and NNW were also computed with 95% confidence intervals. Power analysis was based on the supposition that a mean difference of 0.5 mm and 1 mm in MBL and PD, respectively should be detected at a significance level of 0.05 and a desired study power of at least 80%. It was estimated that a sample size of 70 individuals per group will achieve 95% power with a 0.05 two-sided significance level.

## RESULTS

Mean ages of NW (34.9 years) and NNW (35.2 years) were comparable. All study participants were male. College graduate level education had been attained by 81% NNW and 15% NW. Mean duration of naswar consumption in NW group were 16.1 years. The mean duration of naswar placement in the mouth was 18.3 minutes. Tooth brushing twice a day was reported by 21.1% of NW and 72% of NNW (Table-I).

**Table-I:** General characteristics of the study groups.

	Naswar dippers	Controls
Number of participants (n)	72	70
Mean age in years (range)	34.9 (28-42)	35.2 (25-43)
Graduate education status (%)	15%	81%
Daily frequency	7.3 (2-12)	NA
Mean duration of placement in years (range)	16.1 (8-29)	NA
Mean duration of placement in minutes (range)	18.3 (10-32)	NA
***Daily tooth brushing (%)***		
Once	78.9%	28%
Twice	21.1%	72%

**NA:** Not applicable.

Comparisons between groups for all self-perceived oral symptoms showed statistically significant difference among NW and NNW groups (*p*<0.01) (Table-II). Clinical and radiographic periodontal parameters were significantly worse in NW group as compared to NNW group (*p*<0.01) (Table-III).

**Table-II:** Group comparisons for self-perceived oral symptoms between *naswar* dippers (NW) and control group (NNW).

Characteristics	Group comparison for self-perceived oral symptoms	Yes (n)	No (n)	Odds ratio (95% Confidence interval)	P-value
Pain in teeth	NW (n=72)	61	11	9.5 (6.8-12.3)	<0.01
	NNW (n=70)	5	50		
Pain on chewing	NW (n=72)	57	15	8.6 (7.2-14.9)	<0.01
	NNW (n=70)	7	38		
Bleeding from gums	NW (n=72)	64	8	12.5 (10.1-17.8)	<0.001
	NNW (n=70)	8	47		
Burning sensation in mouth	NW (n=72)	53	19	8.9 (6.3-10.2)	<0.01
	NNW (n=70)	44	11		

**NW:** Naswar dippers, **NNW:** Control group, P-value significance denoted with bold text.

**Table-III:** Means and range of clinical and radiographic periodontal parameters among naswar dippers and control.

Parameters	NW	Control
Number of individuals	72	70
Plaque index (%)	54.2 (50.4-61.3)*	22.4 (13.8-29.5)
Bleeding on probing (%)	57.8 (52.1-63.8)*	8.2 (5.6-13.9)
Probing depth (4-6 mm) (%)	29.7 (22.6-33.5)*	6.3 (2.1-10.4)
Probing depth (>6 mm) (%)	20.4 (11.3-26.6)*	2.7 (1.4-3.8)
Clinical attachment loss (mm)	4.2 (1.4-4.7)*	0.9 (0-2)
Marginal bone loss (mm)	4.9 (1.7-5.2)*	1.8 (1.3-3.5)
Number of missing teeth (%)	6.3 (4-7)*	2.9 (0-4)

* Significantly different from control group (p <0.01)

There was statistically significant effect of daily frequency and mean duration of naswar use on the severity periodontal parameters among NW group. Periodontal parameters were significantly worse among individuals who reported to have been consuming naswar for ≥8 times and ≥20 years (Table-IV).

**Table-IV:** Mean (range) of clinical and radiographic parameters among *naswar* dippers with reference to daily frequency and duration of naswar consumption.

	Naswar dippers (n=72)		

Daily frequency	≤ 4 times	5 to 7 times	≥ 8 times
Plaque index	51.7 (50.1-52.4)	54.7 (51.6-59.6)	57.3 (52.8-61.3)
Bleeding on probing	56.4 (55.2-57.9)	58.8 (54.4-60.7)	59.1 (55.2-61.6)
Probing depth (4-6 mm)	24.8 (23.2-25.7)	32.0 (28.5-33.6)	34.1 (32.4-34.5)
Probing depth (>6 mm)	17.6 (12.4-19.3)	21.7 (18.3-22.6)	23.1 (19.4-24.9)
Clinical attachment loss	3.1 (2.4-3.5)	3.9 (3.3-4.3)	5.6 (4.1-6.0)
Marginal bone loss	4.1 (2.2-5.0)	4.8 (3.6-5.4)	5.7 (4.5-6.3)
Duration	≤ 10 years	11 to 20 years	≥ 21 years
Plaque index	53.8 (51.1-54.6)	54.8 (52.9-58.6)	59.2 (55.6-62.4)
Bleeding on probing	55.6 (54.7-56.3)	56.3 (54.4-58.4)	56.9 (53.1-60.2)
Probing depth (4-6 mm)	24.2 (23.3-26.0)	31.1 (28.5-33.4)	33.8 (32.9-34.6)
Probing depth (>6 mm)	13.4 (12.1-18.8)	22.4 (16.8-23.7)	24.6 (21.3-25.6)
Clinical attachment loss	2.8 (2.7-3.2)	3.3 (2.6-4.4)	4.9 (3.9-5.3)
Marginal bone loss	3.7 (2.9-4.5)	4.4 (3.1-5.9)	5.0 (4.3-6.1)

## DISCUSSION

The potential periodontal health effects of *naswar* have received much less attention. To our knowledge, this is the first study that has compared self-perceived oral symptoms and analysed clinical and radiographic parameters of periodontal health among NW and NNW.

*Naswar* is one of the STP.^13^,^14^ The present findings clearly demonstrated that clinical and radiographic periodontal parameters were significantly high in NW as compared to their respective controls and that a strong association between periodontal injury and *naswar* dipping exists. The results of the present study corroborate with previous studies that demonstrated similar results where the hypothesis was postulated with other STPs.^6^,^15^,^16^ This accounts for the main constituents of naswar containing slaked lime and tobacco have the propensity to enhance the expression of ROS in the periodontal tissues. These chemical species augment inflammation and alveolar bone destruction by reducing endothelial nitric oxide synthase expression and expressing pro-inflammatory cytokines [such as tumor necrosis factor-alpha, interleukin (IL)-6 and IL-1β].^17^-^19^ In addition, nicotine in tobacco has been reported to produce vasodilation in the gingival circulation of periodontal tissues.^10^ This seems to be a possible justification for the enhanced BOP and self-perceived gingival bleeding among NW compared to controls.

Questionnaires are acceptable and reliable assessment tools for evaluating self-reported subjective well-being of individuals.^20^ This study supports our results as self-perceived oral symptoms such as pain on chewing and gingival bleeding were more often reported by NW than controls. Response for burning sensation in the mouth was significantly reported by NW than controls. This may certainly have appeared due to the presence of oral submucous fibrosis (OSF) among NW group. Submucous fibrosis is a precancerous condition characterized by inability to open the mouth due to the stiffening of the oral mucosa and oropharynx, accompanied by burning sensation and blanching in the oral cavity.^21^ Studies have indicated that persistent and long-term habitual tobacco use is a significant risk factor for OSF and oral squamous cell carcinoma.^22^ Since a burning sensation in the mouth was more often reported by NW than controls, it is assumed that OSF may have contributed with the burning sensation in NW dippers.

Factors such as duration and daily frequency of *naswar* placement in the buccal vestibule can also affect the induced oral inflammatory response. Patients recruited in this study reported the duration of *naswar* placement of more than four years in the NW group. It is enticing to hypothesize that consuming *naswar* less frequently and placing these products inside the mouth for shorter duration form less amounts of ROS and hence less amount of periodontal injury. Other important factors that regulate oral health condition are education status and daily self-performed oral home care.^16^ In our study, ~81% of controls had achieved secondary or college graduate level education as compared to NW (15%). Moreover, twice daily toothbrushing was reported by 72% controls, while only 21.1% NW performed twice daily toothbrushing demonstrating poor oral hygiene and greater gingival inflammation. One clarification which can be suggested in this concern is that due to the higher education level, individuals in the control group might be aware of the health risks related to *naswar* use having detrimental effects on periodontal tissues as compared to NW group. Furthermore, it seems that control group (due to higher education status) was more cautious of the fact that plaque control is essential for oral health.^23^

## CONCLUSION

Self-perceived oral symptoms, clinical and radiographic periodontal parameters were worse among NW than NNW. It is suggested that *naswar* dipping should be considered a potential periodontal threat that could have major effects on periodontal tissues.
